# Early Intervention with Highly Condensed Adipose-Derived Stem Cells for Complicated Wounds Following Filler Injections

**DOI:** 10.1007/s00266-016-0636-7

**Published:** 2016-04-21

**Authors:** Joo Hyun Kim, Seong Hoon Park, Byeong Ho Lee, Hii Sun Jeong, Hyun Jin Yang, In Suck Suh

**Affiliations:** Department of Plastic and Reconstructive Surgery, Kangnam Sacred Heart Hospital, College of Medicine, Hallym University Medical Center, 1, Singil-ro, Yeongdeungpo-gu, Seoul, 07441 Korea; Baroil Aesthetic Plastic Surgical Clinic, Seoul, Korea

**Keywords:** Adipose-derived stem cells, Necrosis, Tissue repair, Filler complication

## Abstract

**Background:**

A rise in cosmetic procedures has seen the use of fillers become more prevalent. Complications resulting from use of fillers have prompted introduction of various medical and surgical interventions. Recently, stem cell therapies have become more widely used as a new treatment option for tissue repair and regeneration.

**Methods:**

We utilized adipose-derived stem cells (ASCs) for tissue regeneration in patients with filler-related complications such as necrosis. All 12 patients were treated with ASCs and some patients had additional treatment. After relief of symptoms, wound surface area was compared in terms of pixel numbers and scar condition was evaluated using the Vancouver Scar Scale (VSS).

**Results:**

In general, we achieved satisfactory resolution of filler-related complications in a short period of time without serious side effects. The average number of days from stem cell treatment to symptom relief was 7.3 days. The proportion of wound surface area from photographic record was 4.39 % before treatment, decreasing considerably to 1.01 % following treatment. Last, the VSS showed almost all patients scored below 3, with two patients receiving scores of 7 and 8; the average score was 2.78 (range from 0 to 8).

**Conclusions:**

ASCs are a new treatment option for post-filler injection wounds such as necrosis. Using stem cells, we were able to obtain satisfactory results in a short period of time without complications requiring surgical procedures. We suggest stem cell injections could be used as the first option for treatment of complications from filler injections.

**Level of Evidence V:**

This journal requires that authors assign a level of evidence to each article. For a full description of these Evidence-Based Medicine ratings, please refer to the Table of Contents or the online Instructions to Authors www.springer.com/00266.

## Introduction

Use of soft tissue fillers to restore volume to depressed areas of skin for maintenance of a youthful appearance has been widespread in recent years. Such use is very popular because of its simplicity, low invasiveness, and procedure reversibility, as well as a relatively safe profile. However, as use of fillers has increased, so too has the number of reported complications. These complications, such as vascular compromise and skin necrosis, are often significant and permanent.

Use of stem cells with regenerative potential, such as embryonic or mesenchymal stem cells, has recently emerged as a new form of therapy for wound management. Stem cells are multipotent and possess the ability to self-renew. They are thought to help heal wounds by stimulating angiogenic processes and secreting angiogenic factors; however, the exact mechanisms remain unknown. A newer type of adult stem cell, derived from adipose cells within fat tissue, was discovered at the end of the 20th century. These stem cells are known as adipose-derived stem cells (ASCs) [1].

We have observed many patients with complications following the use of soft tissue fillers. Despite attempts at remediation with various treatments, including dressing with various materials, surgical debridement, skin grafts and local flaps; unsightly skin loss, scarring, and asymmetry still occurred. Therefore, in an effort to maximize rapid recovery and minimize adverse sequelae, we examined the use of stem cell therapy at an early stage of wound development in patients who had received filler injections.

## Materials and Methods

### Patients

Of the 53 patients who visited our clinic for complications following facial filler injections, during the period from 2010 to 2014, we retrospectively analyzed 12 people who had been treated with ASC injection.

These patients, who had filler injections on the glabella or nose, presented with various symptoms including erythema, pustules, skin necrosis, eschar formation, and blindness.

### Patient Review and Treatment Procedure

This study was performed using a medical chart review method that included analyzing demographic data (male/female, mean age), type of filler injection, injection area, symptoms and signs, time interval between filler injection and stem cell treatment, treatment period, remaining complications, and additional secondary treatments. All 12 patients were treated with injections of ASCs and some patients had additional treatment such as antibiotics or steroids. The authors compared wound surface area before treatment and after relief of symptoms related to mild complications (erythema, hypopigmentation, scar, etc.). From photographs, wound surface area was calculated in terms of pixel numbers displayed by the ImageJ program (US National Institutes of Health, Bethesda, MD, USA), with values compared before and after ASC treatment. Scar condition was also evaluated in nine patients (three patients were lost at follow-up) using the Vancouver Scar Scale (VSS).

### Preparation of ASCs for Injection

A harvesting cannula, with a blunt tip attached to a 50-ml Luer-Lok syringe, was inserted into a 3-mm abdominal incision and adipose tissue was harvested from patients’ abdomens by liposuction using a Lipokit (Medikan Inc., Seoul, Korea) to extract stem cells. Harvested fat tissue collected in the syringe was subsequently subjected to adipo-dissociation. First, fat tissue was centrifuged for 4 min at 3500 rpm, resulting in approximately 20 cc of adipose tissue separating into layers, which were then mixed with collagenase Type II (Worthington Industries, Columbus, OH, USA) and liquefied with 20 cc of saline solution. During this process, collagenase Type II is essential for effective recovery of cells from adipose tissue by enzymatic digestion [2]. Adipose tissue and collagenase Type II were then incubated in a Luer-Lok syringe for 30 min at 37 °C using a Maxstem kit (Medikan Inc.) before centrifugation for 3 min at 3500 rpm. Subsequently, 2 cc of solution collected from the bottom fraction was treated with 950 cc of Hartmann solution, and 50 cc of 5 % dextrose saline and washing solution with gentamicin. After three repetitions of this centrifugation and wash procedure, a 2-cc stroma-vascular fraction (SVF) pellet was obtained. Two milliliters of a solution containing ASCs, endothelial cells, endothelial progenitor cells, pericytes, smooth muscle cells, leukocytes, and erythrocytes were divided into 1-ml syringes [3]. This final solution was injected into the lesion at subcutaneous and dermis layers near the wound [4].

After injection of ASCs, ointment-impregnated gauze with topical antibiotics was applied to the raw surface. This dressing was changed at approximately the same time every day until wounds were epithelized. Patients received management for scar prevention using a silicone gel sheet and ointment after total healing.

## Results

**Fig. 1:**
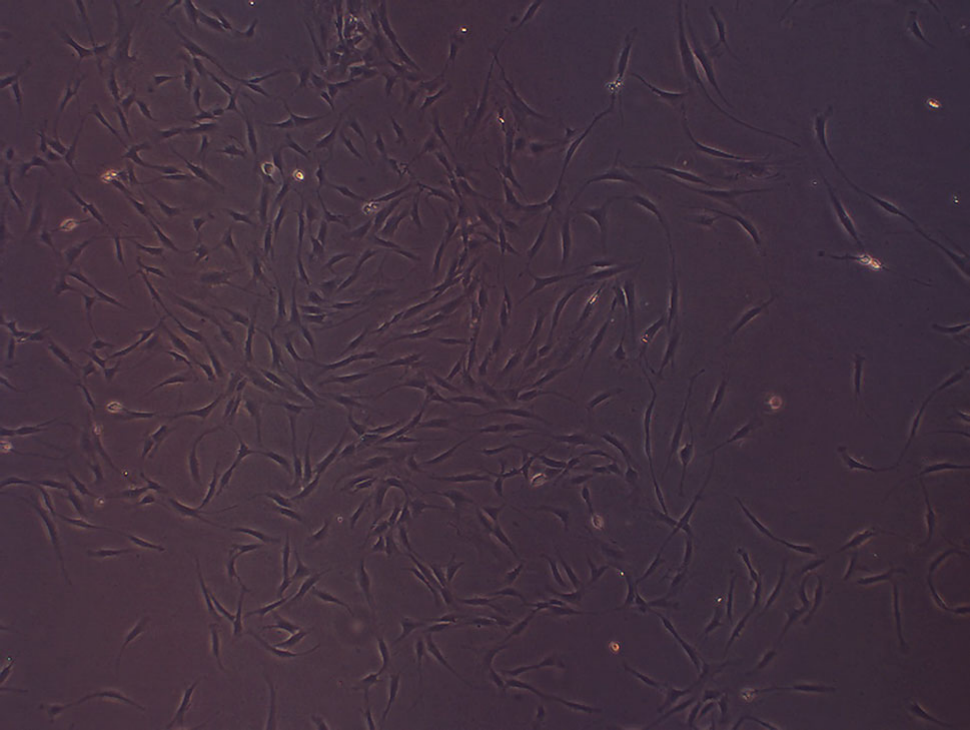
The extracted SVF samples were sent to laboratory for cell counting using a hemocytometer. There were about 1 × 10^5^ cells per 1 ml of SVF

Characteristics of the study population are shown in Table 1. Twelve female patients, average age of 35.6 years (24–52 years), received ASC therapy for adverse reactions to filler injections they had received from 2009 to 2014. Cell counts for SVF samples used in treatments were about 1 × 10^5^ cells per ml, as determined by hemocytometry (Fig. 1).

**Table 1:** Patient and stem cell treatment profile

Case	Age	Filler	Site	Doctor	Symptoms	Filler to stem cell injection interval (days)	Other treatment	Symptom relief after stem cell injection	Complication
1	42	Cutegel	Nose	PS	Necrosis, swelling	7	Debridement, antibiotics	22	Erythema, scar
2	26	Restylane	Nose	PS	Erythema, necrosis	7	Antibiotics	2	Mild erythema
3	35	Unknown	Nose	PS	Erythema, necrosis	3	Antibiotics	5	Mild scar
4	24	Juvederm	Nose, glabella	GP	Necrosis, swelling, blindness	9	Fat injection, antibiotics	4	Mild hypopigmentation
5	39	Unknown	Nose, glabella	Derma	Necrosis, swelling, blindness	5	Antibiotics	F/U loss	F/U loss
6	29	Unknown	Nose, glabella	PS	Erythema, blindness	3	Antibiotics	5	Mild erythema
7	52	Restylane	Nose	Derma	Erythema, scar, contracture	90	Fat injection	7	Mild scar
8	28	Artecoll	Nose, glabella	Non-practitioners	Pustule, necrosis	7	Debridement, antibiotics	10	Mild scar
9	30	Juvederm	Nose	GP	Erythema, necrosis	11	Debridement, antibiotics	8	Mild scar
10	52	Cutegel	Nose	GP	Erythema, necrosis	10	Debridement, antibiotics	F/U loss	Scar, deformity
11	25	Unknown	Nose	Non-practitioners	Erythema, pustule	5	Steroid, antibiotics	3	None
12	45	Unknown	Nose	Non-practitioners	Erythema, scar contracture	3	Composite graft, antibiotics	7	Mild scar

*PS* plastic surgeon, *GP* general practitioner, *Derma* dermatologist, *F/U loss* follow-up loss

All patients had filler injections on the nasal area, with three patients also having had injections on the glabella, which were all separately performed by a total of four plastic surgeons, five general practitioners and dermatologists, and three non-practitioners. Chief complaints from patients were erythema, necrosis, swelling, pustules, and blindness. Necrosis was noted in ten cases, erythema in eight cases, and blindness in three cases. The average interval between filler injection and stem cell treatment for 11 of the 12 patients was 5 days. One of the 12 patients had delayed stem cell treatment, visiting the clinic 3 months after her filler injection, resulting in an overall interval range of 3–90 days and an average of 13.7 days. Antibiotic administration was the most common additional treatment (11 cases). The average number of days it took from stem cell treatment administration to relief of symptoms in nine patients was 7.3 (2–22 days). Three cases could not be assessed because they were not able to be followed up after treatment.

**Fig. 2:**
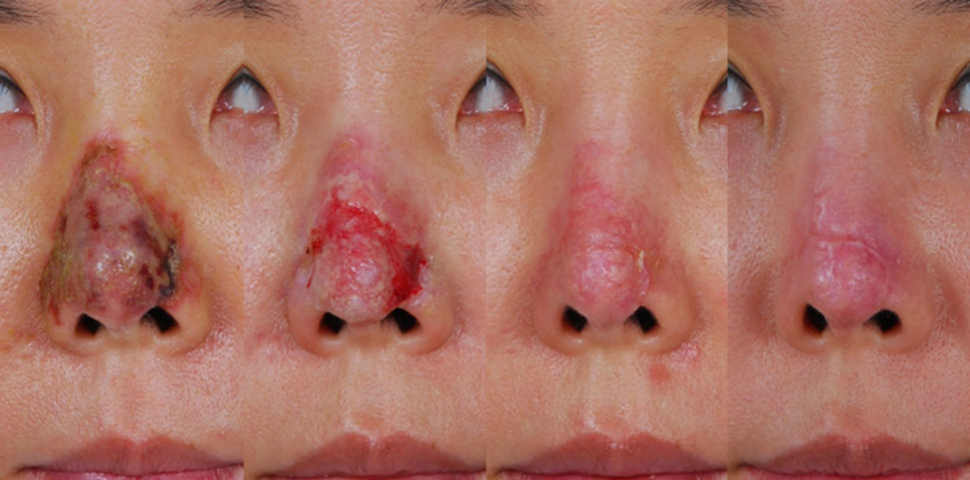
A 42-year-old woman developed skin necrosis of the nose following a filler (Cutegel) injection into the nasal dorsum and tip. Nine days after debridement and stem cell injection, necrotic tissue almost disappeared, and partial raw surface was showing. After 2 months, except for erythema, the wound was healing well. One year after treatment, remnant scar was still showing, but the erythema had decreased considerably

**Fig. 3:**
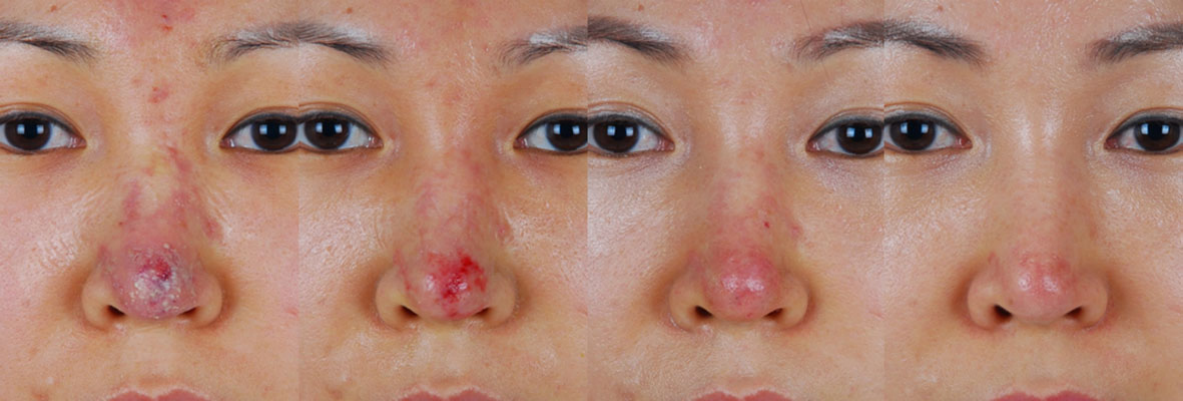
A 35-year-old female developed necrotic changes following a filler (unknown) injection into the nasal dorsum and tip. Three days after the filler injection, the patient came to the clinic with erythema and necrosis. Stem cell therapy was started, and after 3 days, necrotic tissue disappeared and partial raw surface was showing on the nasal tip. One week after stem cell injection, mild erythema remained, and after 7 weeks, the wound had healed into normal skin

**Fig. 4:**
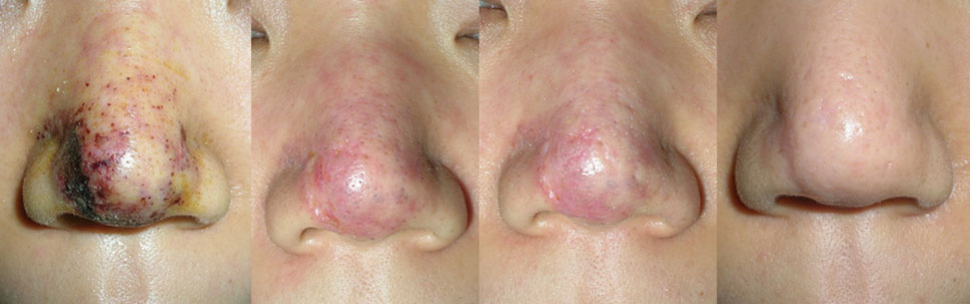
A 23-year-old female patient came to hospital suffering from pain, edema, skin necrosis, and pus discharge following a filler injection (Artecoll) into the forehead, nasal ridge, and tip. Seven days after debridement and stem cell injection, the wound had totally healed except for mild erythema and scarring. One month later, the scarring and erythema showed no considerable change, but after 6 months, no complications were shown

We observed seven cases of mild scarring, three cases of erythema, and one case of hypopigmentation. However, all wounds healed in a short period of time without serious side effects (Figs. 2, 3, 4). To minimize remaining complications, five patients received a secondary stem cell treatment and one patient required a composite graft for nostril notching. To achieve objective comparison between remaining lesions and original wound sites, pixels were calculated from photographs of the lesion, as previously described. Briefly, ImageJ software was used, by two unrelated physicians, to calculate the number of pixels of evident wound and complication sites before and 6 months after treatment. The proportion of wound surface area to total photographic pixels was 4.39 % before treatment, decreasing substantially to 1.01 % after treatment with ASCs (Table 2). Using the Vancouver Scar Scale (VSS), two physicians evaluated scar condition in nine patients who completed the study at 6 months post-treatment.

**Table 2:** Comparison of areas between wound site before treatment and after treatment

Patient	Pixels of wound or complication area/total pixels of photograph (%)	
	Pre-treatment	Post-treatment (6 months)
1	15.83	0.37
2	0.34	0.29
3	6.78	2.95
4	3.33	0.61
8	3.16	0.26
9	2.28	1.35
11	2.46	1.46
12	0.91	0.79
Average	4.39	1.01

The VSS consists of four parameters: pigmentation, vascularity, pliability, and height. The highest score on this scale is 13, indicating the worst condition of a scar, whereas a lower score indicates an improved scar condition. Almost all of the patients scored below 3, with two patients receiving scores of 7 and 8; the average score was 2.78 (range from 0 to 8).

## Discussion

Since the first autologous fat injection for face volume restoration was performed [5], many fillers (e.g., bovine collagen [Zyderm, Zyplast], Restylane, etc.) have been developed and approved by the Food and Drug Administration. As corresponding usage of fillers has increased, reported cases of adverse effects have also increased; all fillers can carry a degree of risk of mild to severe complications. Mild adverse effects include swelling, erythema, and nodules; moderate effects include infection and scars, whereas serious adverse effects include blanching resulting from vascular compromise and necrosis. The most severe complication from filler injections is skin necrosis. A few hypotheses explain mechanisms underlying necrosis, such as allergic reaction (hypersensitivity), external compression, and intra-arterial injection; however, artery embolism induced by intra-arterial injection is the most accepted theory. Whereas most complications are transient, more severe adverse events, including necrosis, can leave patients with long-lasting or permanent aesthetic and functional problems [6].

Although it is important to treat complications when they are observed, prevention is a far better way to deal with complications. When injecting filler, rapid flow rate and high volume are risk factors for increased side effects. Therefore, adequate amounts of filler should be injected slowly to get low pressure. Small-caliber and blunt needles should be used, while aspiration prior to injection is necessary to reduce the rate of tissue necrosis. Despite all efforts, if an unavoidable complication occurs, appropriate treatment should be performed as soon as possible.

According to the treatment algorithm for complications after filler injections [7], treatments vary depending on symptoms. Moreover, treatment is decidedly different for wounds, which are defined as a disruption of normal anatomic structure and functional integrity of the skin. Usually, debridement, antibiotic therapy, and hyperbaric oxygen therapy are carried out for wounds extending deep into the dermis that also present with necrotic tissue. However, when these repair strategies are ineffective, other methods, such as skin grafts, should be considered. These treatments can result in protracted healing and sequelae, such as scars, contractures, and pigmentation, according to our experience. As wound healing has been linked to release of cytokines, chemokines, and growth factors, stem cell therapy is emerging as a new treatment of great interest. Further, since debridement, skin grafts and similar treatments are highly invasive and extremely destructive, our institution has been applying stem cell therapies more frequently to facilitate wound healing. Because of their multipotency, ASCs can be used in a wide variety of clinical applications such as diabetic or chronic radiation ulcers, which are difficult to resolve.

In addition, we applied ASCs in blindness cases. In our research, blindness was reported in three cases with symptoms improving after ASCs were administered at the retrobulbar area. General treatment for blindness includes ophthalmologic evaluations such as consultation and angiography. And aspirin administration, hyaluronidase injection, and oxygen therapy can be possible treatments. But our results indicate when early detection of blindness and rapid application of ASCs is possible, we suggest ASCs as an optional treatment. Compared with other available treatments, ASCs are generally harmless. Further, considering all three patients showed a favorable response to the treatment, ASC application can be a useful method for treating symptomatic blindness. But, further evaluation is necessary.

**Fig. 5:**
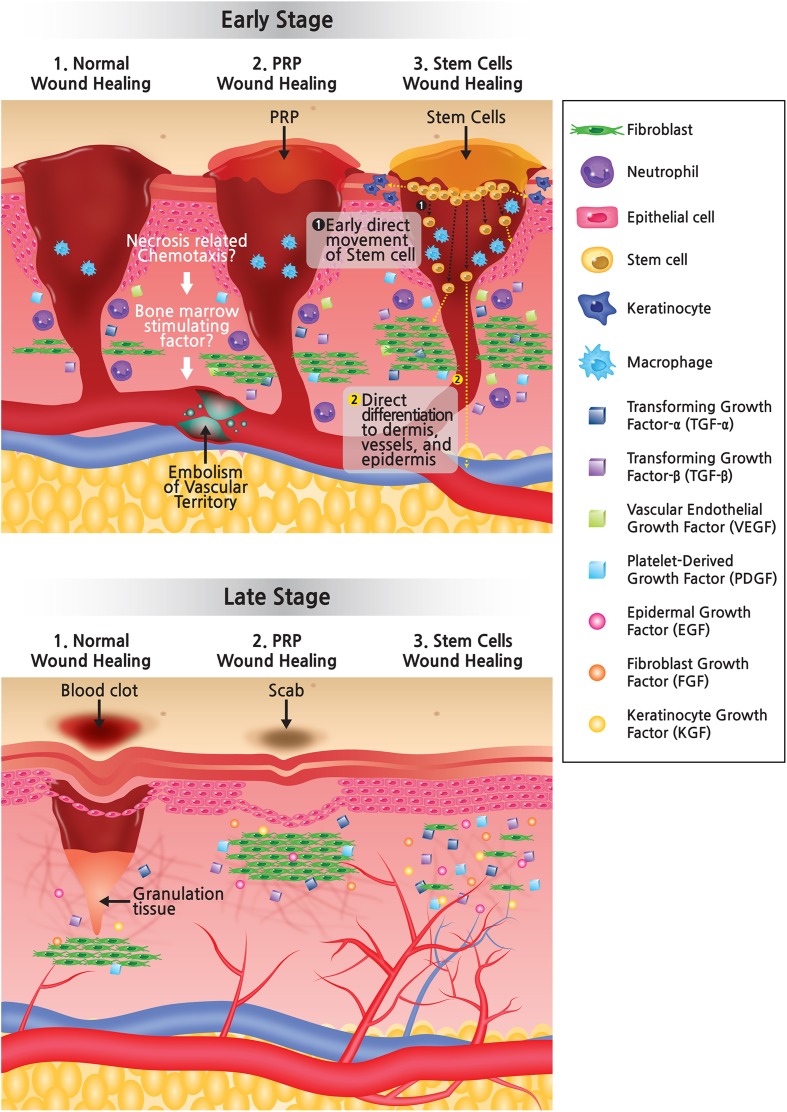
As compared with normal wound healing or PRP wound healing process, in the early stage of wound healing, early direct movement of the stem cells allows direct differentiation to the dermis, vessels, and epidermis, secreting various growth factors. As a result, almost perfect wound healing can occur without blood clot and scab in the late stage

Most patients treated in this center were injured at a level deeper than the subcutaneous layer and indirect comparison was performed due to lack of a control group. According to one study investigating wound healing with bFGF treatment in second-degree burn patients, wound healing in the bFGF group took 12.0 ± 2.2 days compared to 15.0 ± 2.7 days for control groups using ointment and gauze [8]. Additionally, Kazakos et al. suggested treatment duration of soft tissue wounds using autologous PRP took 21.26 ± 1.35 days on average among 27 patients applying PRP [9]. In contrast, we were able to successfully relieve symptoms without major complications in a reasonably short time span (average of 7.3 days), although results varied depending on severity of symptoms at the patient’s first presentation. Compared with normal wound healing or a PRP (platelet-rich plasma) wound healing process, early direct movement of stem cells allows wound healing without blood clots or scab formation through direct differentiation to dermis, vessels, and epidermis (Fig. 5).

In this article, there was no control group treated for filler complications with other methods, making a direct comparison of treatment periods difficult. Also, although most patients displayed necrosis when ASC treatment commenced, variation in the extent of necrosis resulted in differences of duration for symptom relief, scar condition, and post-treatment results. Accordingly, severity of necrosis is an important factor for competent treatment. Our study is also limited by a relatively small group of 12 patients. If there had been more patients alongside a control group with general dressing, a more objective and definitive result could be assumed. Nevertheless, with our own results and what is known from other studies on this topic, we can conclude that starting stem cell therapy early is helpful for symptom relief in 12 patients with adverse reactions to filler injections. In addition, when comparing the number of pixels between the two sites—before treatment and complications remaining after treatment—and evaluating scar condition with the VSS, we found that stem cell therapy objectively results in reduced scar formation without major complications. In the future, an appropriate concentration of stem cells should be administered according to wound severity and based on research regarding exact stem cell mechanisms.

## Conclusions

Filler injections are seen as an easy way to restore volume to a depressed area of the skin. However, numerous complications following filler injections have been reported, with various treatments introduced to address these issues. ASCs are a new treatment option for post-filler injection wounds such as necrosis, erythema, and scar formation. Using stem cells in a limited study, we were able to obtain satisfactory results in a short period of time without any sequelae requiring surgical procedures. Before ASCs were introduced, the treatment of choice for small defects was surgical intervention according to a reconstruction ladder. Our results suggest stem cell injections could be used as the first option for treatment of complications from filler injections.

In conclusions, early-condensed adipose-derived stem cell injections may resolve various complications, such as necrosis following a filler injection to the face, by promoting rapid re-epithelization, angiogenesis, and regeneration of soft tissues.
